# Predicting Disability after Ischemic Stroke Based on Comorbidity Index and Stroke Severity—From the Virtual International Stroke Trials Archive-Acute Collaboration

**DOI:** 10.3389/fneur.2017.00192

**Published:** 2017-05-19

**Authors:** Thanh G. Phan, Benjamin B. Clissold, Henry Ma, John Van Ly, Velandai Srikanth

**Affiliations:** ^1^Stroke Unit, Monash Health and Stroke and Aging Research Group, Monash University, Melbourne, VIC, Australia

**Keywords:** stroke, disability evaluation, Charlson comorbidity score, prediction, National Institutes of Health Stroke Scale scores

## Abstract

**Background and aim:**

The availability and access of hospital administrative data [coding for Charlson comorbidity index (CCI)] in large data form has resulted in a surge of interest in using this information to predict mortality from stroke. The aims of this study were to determine the minimum clinical data set to be included in models for predicting disability after ischemic stroke adjusting for CCI and clinical variables and to evaluate the impact of CCI on prediction of outcome.

**Method:**

We leverage anonymized clinical trial data in the Virtual International Stroke Trials Archive. This repository contains prospective data on stroke severity and outcome. The inclusion criteria were patients with available stroke severity score such as National Institutes of Health Stroke Scale (NIHSS), imaging data, and outcome disability score such as 90-day Rankin Scale. We calculate CCI based on comorbidity data in this data set. For logistic regression, we used these calibration statistics: Nagelkerke generalised *R*^2^ and Brier score; and for discrimination we used: area under the receiver operating characteristics curve (AUC) and integrated discrimination improvement (IDI). The IDI was used to evaluate improvement in disability prediction above baseline model containing age, sex, and CCI.

**Results:**

The clinical data among 5,206 patients (55% males) were as follows: mean age 69 ± 13 years, CCI 4.2 ± 0.8, and median NIHSS of 12 (IQR 8, 17) on admission and 9 (IQR 5, 15) at 24 h. In Model 2, adding admission NIHSS to the baseline model improved AUC from 0.67 (95% CI 0.65–0.68) to 0.79 (95% CI 0.78–0.81). In Model 3, adding 24-h NIHSS to the baseline model resulted in substantial improvement in AUC to 0.90 (95% CI 0.89–0.91) and increased IDI by 0.23 (95% CI 0.22–0.24). Adding the variable recombinant tissue plasminogen activator did not result in a further change in AUC or IDI to this regression model. In Model 3, the variable NIHSS at 24 h explains 87.3% of the variance of Model 3, follow by age (8.5%), comorbidity (3.7%), and male sex (0.5%).

**Conclusion:**

Our results suggest that prediction of disability after ischemic stroke should at least include 24-h NIHSS and age. The variable CCI is less important for prediction of disability in this data set.

## Introduction

Stroke is a leading cause of disability worldwide and results in significant economic and societal cost. Data from Global Burden of Disease 2015 show that stroke and ischemic heart disease accounted for 15.2 million deaths worldwide or approximately 85.1% (84.7–85.5) of all deaths due to cardiovascular disease (1). Hospital administrators across the world (2), international consortium (3), the media, and the publics (4) (http://www.abc.net.au/news/2013-12-05/new-report-highlights-hospital-mortality-rates/5135858) are concerned about hospital performance with regards to outcome after stroke. Various groups including those from health-care information company, Dr Foster, have considered the Charlson comorbidity index (CCI) as a way to measure hospital performance. This has been done in the hope that better measurements would lead to improvement in care (2, 5, 6). The CCI acted as a weight in the calculation of the standardized hospital mortality rate. Within Australia, there are several groups that used comorbidity index for monitoring hospital performance (7). Some Australian hospitals have been named for “higher than expected mortality rate” for stroke and heart attacks (http://www.abc.net.au/news/2013-12-05/new-report-highlights-hospital-mortality-rates/5135858).

The CCI was conceived as a method for classifying prognostic comorbidity in longitudinal studies (8). It is a weighted index of comorbid conditions and is extracted from data entered into hospital medical records (9). This action is usually performed by administrative rather than clinical staff. Investigators have also developed method to collect CCI from electronic medical records (10). The earlier optimism of different types of comorbidity indices, as risk adjustment for the prediction of mortality after stroke (11, 12), has been recently questioned by several investigators (13–15) including the team from Dr Foster in 2016 (16). This may have occurred because CCI captures comorbid conditions in general but contains only a small component for capturing the effects of stroke.

The value of the covariate CCI in the prediction of disability after ischemic stroke is less well understood and is the subject of this study. Previous investigators have described that patients with low CCI had better outcome at discharge than those with high CCI (17, 18). However, these authors had not adjusted for stroke severity or other variables such as thrombolytic therapy with recombinant tissue plasminogen activator (rTPA). In a small study (*n* = 133), investigators have suggested that women with higher CCI have greater disability after stroke after adjusting for stroke severity (19). Findings from this study (20) and the earlier study, from the same group, (18) on CCI had been recently incorporated into a proposed a neuroeconomic approach towards decision making on rTPA therapy (21). In light of the multiple uses of CCI, the aims of this study were to determine the minimum clinical data set to be included in model predicting disability after ischemic stroke after adjusting for CCI and clinical variables and to evaluate the impact of CCI on prediction of disability outcome. To achieve this purpose, we have identified clinical trial repository such as the Virtual International Stroke Trials Archive (VISTA) (21) as having data on stroke deficit, comorbidity, and outcome.

## Materials and Methods

This study used data from the VISTA archives of stroke clinical trials (21). The methods used for this study have been described in a related paper on prediction of mortality from stroke (15). The following terms were used to search the repository for this data set: imaging data—Alberta Stroke Program Early CT Score (ASPECTS) on CT scan (the ASPECTS assesses the extent of ischemia over 10 regions of the middle cerebral artery territory); stroke deficit—National Institutes of Health Stroke Scale (NIHSS) on admission and at 24 h; physiological variables on admission (systolic blood pressure, blood glucose level); demographic data (age, sex); stroke risk factors and comorbidities (including but not limited to hypertension, diabetes, atrial fibrillation, degree of liver impairment, degree of renal impairment); thrombolysis treatment with rTPA (22); and outcome data—modified Rankin outcome within 90 days of stroke. Rankin scale of 0 signifies no symptom and 6 signifies death. The modified Rankin scale (mRS) of 2 equates to mild disability and mRS 3 equates to moderate disability. In this study, the primary outcome is disability at 90 days. We defined disability as mRS between 3 and 6.

### Charlson Comorbidity Coding in VISTA

The CCI is calculated from administrative coding of medical record (9). Variables given weight of 1 in CCI included myocardial infarct, congestive heart failure, peripheral vascular disease, cerebrovascular disease, dementia, chronic pulmonary disease, connective tissue disease, ulcer disease, mild liver disease, and diabetes. Variables given weight of 2 in CCI included hemiplegia, moderate or severe renal disease, diabetes with end-organ damage, any tumor, leukemia, and lymphoma. Variables given weight of 3 in CCI included moderate or severe liver disease. Variables given weight of 4 in CCI included metastatic solid tumor and advanced immunodeficiency syndrome.

The variable diabetes in the CCI was assigned a coding of 2 because stroke can be considered to represent “diabetes with end organ damage.” To code motor deficit, we used NIHSS ≥ 6 (the minimum NIHSS in this VISTA data set was 6). For the purpose of analysis, the combined score of comorbid conditions was used.

### Statistical Analysis

Correlation between stroke severity and CCI was performed using Spearman method. The optimal was derived by successively adding variables to the logistic regression models. Statistics for measuring model performance (model discrimination and calibration) are described below.

Model 1 = age + sex + CCI.
Model 2 = Model 1 + NIHSS on admission (baseline).
Model 3 = Model 1 + NIHSS at 24 h (removing NIHSS on admission).
Model 4 = Model 3 + rTPA.
Model 5 = Model 2 + rTPA.
Model 6 = Model 3 + rTPA + physiological variables, risk factor, and imaging data, including systolic blood pressure, serum glucose level, hypertension, atrial fibrillation, and ASPECTS score.

### Assessing Model Discrimination

**Figure 1:**
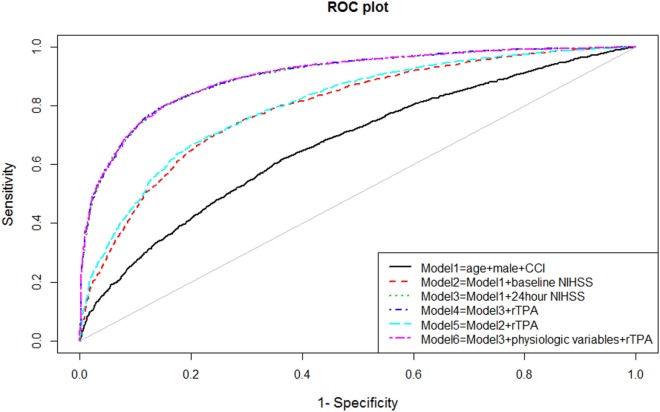
**Receiver operating characteristic (ROC) curves for models of disability**.

The ability of the model to discriminate between those with and without disability at 90 days after stroke was performed by measuring the areas under the receiver operating characteristic (ROC, see Figure 1) curve (AUC). Measurement of model discrimination is different from model calibration (expanded below). Investigators have pointed out that it is not possible to achieve perfection in both calibration and discrimination. A model can have excellent discrimination between two groups but poor calibration (23).

### Model Calibration

Model calibration refers to methods that measure distance between predicted and observed probability, with the minimal distance between the two points describing close matching between predicted and observed probability. We performed calibration by using several complementary methods, namely the Brier score (24) and Nagelkerke generalized *R*^2^ (25). The Brier score is a cost function that measures the mean square difference between the predicted probability and the observed binary outcome. The Nagelkerke generalized *R*^2^ has often been taken to have same meaning as the *R*^2^ in ordinary least square regression (variance of the model explained by the predictors), but the derivation of generalized *R*^2^ is rather different from that for *R*^2^. The generalized *R*^2^ is formulated as the fraction of the log likelihood explained by the predictors (adjusted between 0 and 1). A well-calibrated model has low Brier score and a high generalized *R*^2^ value.

### Measuring Improvement in Regression Models

The AUC is good at discrimination but is not as well suited to detecting difference in discrimination between models due to its low sensitivity for detecting such change (26). With this in mind, the net reclassification improvement (NRI) and integrated discrimination improvement (IDI) have been proposed to detect difference in model discrimination (27). The NRI is the percentage reclassification for the risk categories and included both up and down. The maximal value of NRI is 200%. The IDI is the mean difference in predicted probabilities between new and old regression models (constructed from cases with disease and without disease). The NRI and IDI are expressed here as fractions and can be converted to percentage by multiplying 100. The AUC, continuous NRI, and IDI were performed using *PredictABEL* (R Statistical Foundation, version 3.2.0) (28).

### Hierarchical Partition

**Figure 2:**
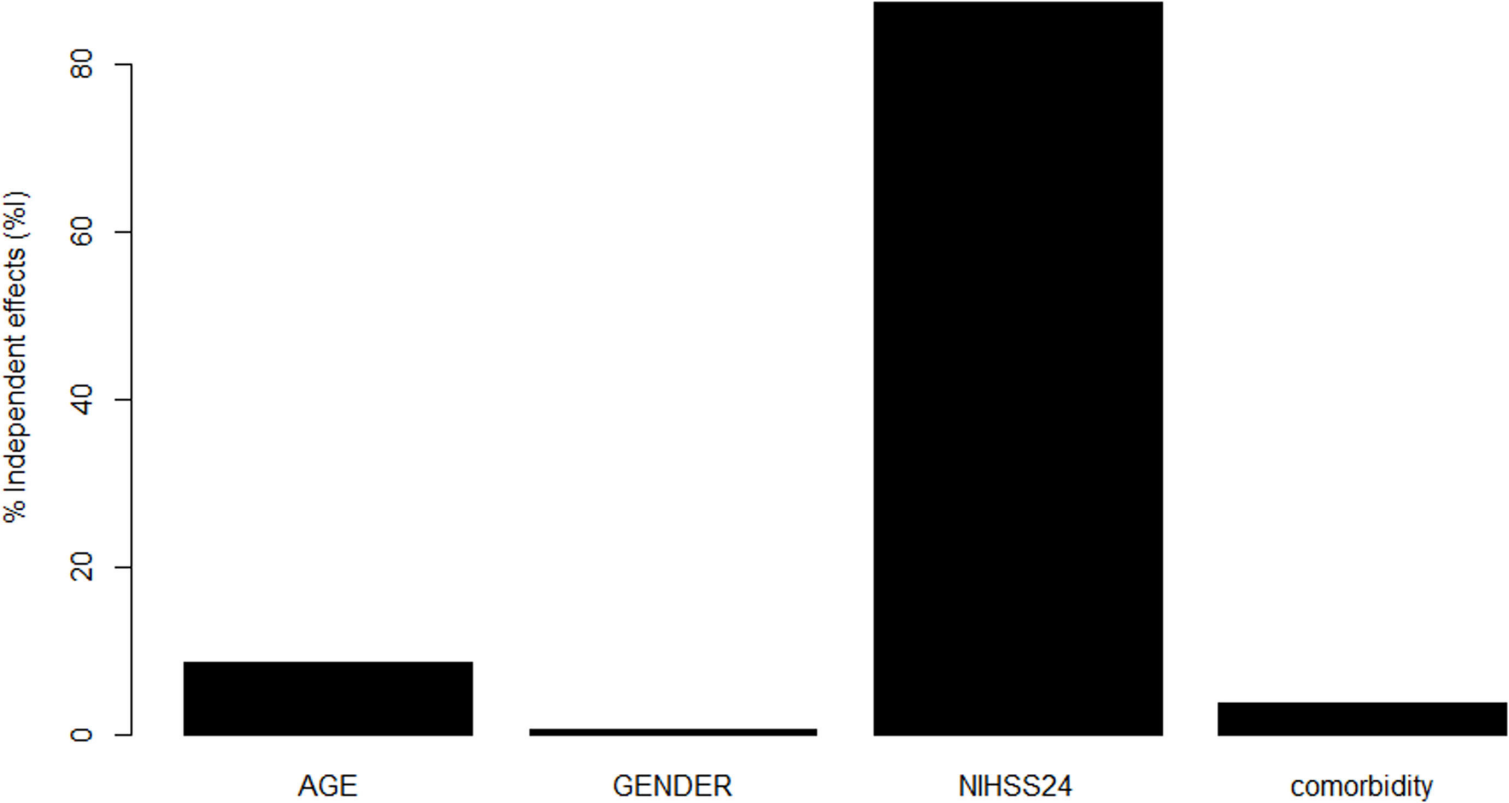
**Contribution of variables in Model 3 to prediction**. National Institutes of Health Stroke Scale (NIHSS) at 24 h made the greatest contribution toward the pseudo *R*^2^ for Model 3, followed by age, comorbidity, and male sex.

Once we have obtained the optimal model, we used hierarchical partition method to find the contribution of important predictors to that model (see Figure 2) (29). The hierarchical partition method attempts to partition the goodness of fit of the models (hier.part package, R Statistical Foundation, version 3.2.0).

## Results

This study consists of 5,206 patients with mean age 68.8 ± 12.5 with 55% male sex, 54.5% ever-smokers, 73.6% with hypertension, 23% with diabetes, and 26% with atrial fibrillation. The median NIHSS at baseline was 12 (IQR 8, 17) and at 24 h was 9 (IQR 5, 15). The comorbidity score (based on admission NIHSS ≥ 6) was 4.2 ± 0.84. The proportion of patients with modified Rankin disability scale >2 at 90 days was 45%. The mortality at day 90 was 15%. In this analysis, 1,807 patients had ASPECTS score recorded. The mean ASPECTS score was 9.8 ± 0.8 (a score of 10 indicates no ischemic involvement of the MCA territory, and score of 0 indicates total involvement).

Univariable analyses showed statistically significant relationships between disability and NIHSS at admission and at 24 h, age, systolic blood pressure, atrial fibrillation, and the use of rTPA. The correlation between comorbidity and baseline NIHSS was 0.09 (*p* < 0.001) and between comorbidity and 24 h NIHSS was 0.11 (*p* < 0.001). The multivariable models for disability and their associated AUC, Brier score, and generalized *R*^2^ are displayed in Table 1. The baseline model (Model 1: age, male sex, and comorbidity index) had an AUC of 0.67 (95% CI 0.65–0.68); Model 2 (Model 1 and NIHSS on admission) had AUC 0.79 (95% CI 0.78–0.81); Model 3 (Model 1 and NIHSS at 24 h) had AUC 0.90 (95% CI 0.89–0.91); and Model 4 (Model 3 and rTPA) had AUC 0.90 (95% CI 0.89–0.91). When physiological variables were added to the multivariable analyses, systolic blood pressure and presence of atrial fibrillation did not remain in the model, and Model 6 consisted of 24-h NIHSS, age, rTPA and serum glucose, and AUC 0.90 (95% CI 0.89–0.91). The plots of these ROC curves are displayed in Figure 1.

**Table 1:** **Models of disability outcome at 90 days**.

	Equation	Nagelkerke *R*^2^	Brier score	AUC	Comparisons	NRI	IDI
Model 1	Comorbidity + age + sex	0.11	0.23	0.67 (0.65–0.68)			
Model 2	Model 1 + NIHSS baseline	0.32	0.18	0.79 (0.78–0.81)	Model 2–Model 1	0.82 (0.77–0.87), *p* < 0.001	0.17 (0.16–0.18), *p* < 0.001
Model 3	Model 1 + NIHSS 24 h	0.59	0.13	0.90 (0.89–0.91)	Model 3–Model2	1.16 (1.12–1.21), *p* < 0.001	0.23 (0.22–0.24), *p* < 0.001
Model 4	Model 1 + NIHSS 24 h + rTPA	0.59	0.13	0.90 (0.89–0.91)	Model 4–Model 3	0.07 (0.01–0.12), *p* = 0.02	0.001 (0.00–0.002), *p* = 0.04
Model 5	Model 2 + rTPA	0.34	0.18	0.80 (0.79–0.81)	Model 5–Model 3	−1.09 (−1.14 to −1.04), *p* < 0.001	−0.21 (−0.22 to −0.19), *p* < 0.001
Model 6	Model 3 + rTPA + physiologic variables	0.63	0.11	0.90 (0.89–0.91)	Model 6–Model 3	0.008 (0.02–0.14), *p* = 0.005	0.002 (0.00–0.004), *p* = 0.003
Model 7	Age + NIHSS 24 h	0.58	0.13	0.90 (0.89–0.91)	Model 7–Model 3	−0.116 (−0.174 to −0.057), *p* < 0.001	−0.002 (−0.003 to −0.00005), *p* = 0.007

*AUC, area under the receiver operating characteristics curve; NRI, net reclassification improvement; IDI, integrated discrimination improvement; NIHSS, National Institutes of Health Stroke Scale; rTPA, recombinant tissue plasminogen activator*.

Model 2 was an improvement on Model 1 with NRI 0.82 (95% CI 0.77–0.87) and IDI 0.17 (95% CI 0.16–0.18) (Table 1). There was statistically significant difference between Models 3 and 2 with NRI [1.16 (95% CI 1.12–1.21)] and IDI [0.23 (95% CI 0.22–0.24)]. There was statistically significant difference between Model 4 and 3, but the small difference in NRI [0.07 (95% CI 0.01–0.12)] and IDI [0.001 (95% CI 0.00–0.002)] suggest that this was not clinically significant. Model 5 (Model 2 and rTPA) was inferior to Model 3 in terms of AUC [0.80 (95% CI 0.79–0.81)], NRI [1.09 (95% CI −1.14 to −1.04)] and IDI [−0.21 (95% CI −0.22 to −0.19)]. In terms of calibration, the increased generalized *R*^2^ and decreased Brier score values paralleled improvement in AUC from Model 1 to Model 3. The calibration metric for Model 5 was lower than for Model 3.

Our results suggest that the Model 3 was the optimal model with the minimum variables to be collected in addition to CCI (age, sex, and 24-h NIHSS). Next, we used hierarchical partition method to explore the key drivers of Model 3 (Figure 2). The variable NIHSS explains 87.3% of the variance, follow by age (8.5%), comorbidity (3.7%), and male sex (0.5%). By using this analysis, we repeat a modification to Model 3 with covariates NIHSS at 24 h and age but without the covariates CCI and male sex. The new Model 3 is comparable to the original Model 3 (see Table 1).

## Discussion

The key findings in this study were the importance of a stroke severity score measured at 24 h in prediction of disability following ischemic stroke. This covariate is superior to the baseline NIHSS if the aim of the prediction model is to define outcome after the initial phase of stroke (16). The covariate, CCI, made a very small contribution to prediction models when used in conjunction with stroke severity as covariate. By systematically evaluating the clinical variables, our study was able to put a limit on the minimum set of variables needed for valid prediction of disability following ischemic stroke. Our study cautions the use of CCI as the main variable for predicting disability following ischemic stroke, monitoring outcome (3), or making decision on acute therapy (20).

In earlier exploration on CCI and stroke, investigators did not adjust for stroke severity as we have done here. This was the case in the Veteran Affairs study (*n* = 960) (17). Along with comorbidity, stroke severity was included in the model in a smaller study (*n* = 133) (19). These studies did not explore the impact on the model using different statistics of model fit and calibration as we have done here. There are two other studies that included stroke severity in the model for disability; these studies had sample sizes less than 614 (16, 30). Stroke severity was defined in those studies according to the baseline NIHSS (16) and Scandinavian Stroke Scale (30). The timing of the assessment of the Scandinavian Stroke Scale was uncertain in the latter study. As such, those studies could not provide contrast between baseline and have 24-h stroke severity data. On its own, CCI discriminates disability after stroke poorly in this study. We took this further by exploring the key drivers within each model with hierarchical partition of the variables. This type of analysis showed that the covariate, CCI, contributed little to the model relative to the covariate, NIHSS at 24 h. The model only discriminates well when the covariate, CCI, was used in conjunction with stroke severity measured at 24 h. This result is consistent with the dynamic concept of the ischemic penumbra. In some cases, reperfusion therapy or delayed spontaneous spectacular shrinking deficit (reperfusion) may lead to good clinical outcome (31, 32). Consequently, measuring stroke severity after therapeutic or spontaneous reperfusion provides a better indicator of final outcome.

We acknowledge that the use of data at 24 h is not helpful for making therapeutic decision on drug such as rTPA. The intention of developing prediction models based on 24-h data is to help the patient and family regarding prognostication on the disability outcome and monitoring of outcome. Our finding that the variable rTPA does not remain in the model when the 24-h NIHSS is used should not be misinterpreted as demonstrating that rTPA is not a predictor of outcome. This model is performed using information at 24 h after stroke onset and after drugs such as rTPA have done their job in rescuing ischemic tissue. This argument on the importance of rTPA is based on the difference between Model 5 and Model 2, where rTPA remains in the model when the baseline NIHSS is used instead of the 24-h NIHSS.

The strength of our study was the use of different statistics for measuring model performance. These metrics for discrimination and calibration provide different evaluation of model performance (23). This effect can be seen in the comparison between Models 2 and 1. These metrics show that a large number of cases (79.0%) would be reclassified if stroke severity is added to the baseline model. Models 3, 4, and 6 have equivalent discrimination and would be classified as having outstanding discrimination (AUC ≥ 0.90) using the guidelines of Hosmer and Lemeshow (33). Model 4 was not preferred as it differed from Model 3 based on NRI but not IDI. As such we considered Models 3 and 4 to be equivalent. When Models 6 and 3 are compared, a small number of cases (10.7%) would be reclassified based on NRI. The IDI between Models 6 and 3 was small, and thus we considered that a very small gain would be obtained by having a more complex model or in practical terms collecting more data. Our finding reaffirmed previous findings on the role of these physiological variables in outcome prediction (34). However, the difference between these two models was small based on the IDI (35). We can propose that the minimum data set would include comorbidity, age, sex, and stroke severity at 24 h (Model 3). Since stroke severity score is not routinely available in administrative data sets, such a model would require prospective recording of stroke severity score in a separate column (of the administrative data set) for extraction. While Model 6 is marginally better than Model 3, its use would require extraction of clinical information beyond those available in routine administrative data.

Our approaches have several limitations. The sample used here come from clinical trial repository and as such does not fully reflect the range of patients seen in hospitals. For example, patients with mild and very severe stroke were not included in this study. Patients with the most severe strokes and dementia are important as they may have multiple comorbidity and are likely to be discharged to nursing home. A potential criticism is that patients who died within 24 h are not included because those patients do not have 24-h NIHSS. The number of those cases with such scenario was reassuringly only five cases. With respect to the real world data, the NIHSS on admission of the patients in this cohort was slightly higher than observational studies by the Get With The Guidelines (13) and Global Comparator Stroke GOAL investigators (16).

The estimation of the comorbidity index in our analysis is also not identical with that derived from usual hospital administrative data sets. It is true that patients in stroke clinical trials do not have significant complex comorbidities such as severe liver failure, terminal malignancy, dementia, or advance immunodeficiency syndrome (9). The exclusion criteria used in stroke trials would have excluded them. On the other hand, patients involved in acute stroke trials are more likely to come from tertiary hospitals where cases with higher stroke severity score are preferentially treated compared to small community hospitals. These limitations could have impacted on the contribution of comorbidities to the prediction of disability in this study. We believe that this is less likely since the comorbidity in our cohort is similar to those in the Veteran Affairs study (17) and Tennessee’s statewide Medicaid managed care program (36). In spite of these limitations, the VISTA data set has several advantages above hospital registry data. Coding of data in hospital administrative data sets may be plagued by inaccuracy in coding of comorbidity (37). To obtain clinical data on stroke severity, several hospitals that participated in the Global Comparators Stroke GOAL (internal project facilitated by Dr Foster) measured NIHSS at baseline for 2 months (16). The measurement of stroke severity score for only 2 months was likely to be due to cost associated with long-term prospective collection of such data. One potential criticism of using NIHSS and CCI in this analysis is that both metrics measured motor deficits. However, all patients received the same motor score in the CCI as such there was no redundancy in the data. We have also analyzed the correlation between these variables and found them to be low. Finally, we have used the term prediction in this article rather loosely. Strictly, it should be used when we validate it in an independent data set. We are not aware of other prospectively collected data set with available information on both baseline and 24-h stroke severity score. We would welcome any such collaboration to validate the model.

### Conclusion

We have applied a systematic approach to finding the minimum clinical data set for reliably determining disability following ischemic stroke onset. The CCI should not be used as it contributes only a small part of logistic model. By contrast, stroke severity score at 24 h is the most important variable.

## Ethics Statement

The VISTA data were collated from deidentified randomized control trials. In each trials, patients had to sign written consent.

## Author Contributions

Study concept and design; drafting of manuscript: TP, BC, HM, JL, and VS. Acquisition of data: TP and VISTA-Acute Collaborators. Analysis and interpretation of data: TP and BC.

## Conflict of Interest Statement

TP reports receiving honoraria as speakers for Genzyme, Boehringer Ingelheim, and Bayer. He is on the advisory board for Genzyme on Fabry Disease. The other authors declare no conflict of interest. The reviewer, CI, and handling editor declared their shared affiliation, and the handling editor states that the process nevertheless met the standards of a fair and objective review.

## Vista-Acute Steering Committee

K. R. Lees (Chair), A. Alexandrov, P. M. Bath, E. Bluhmki, N. Bornstein, C. Chen, L. Claesson, S. M. Davis, G. Donnan, H. C. Diener, M. Fisher, M. Ginsberg, B. Gregson, J. Grotta, W. Hacke, M. G. Hennerici, M. Hommel, M. Kaste, P. Lyden, J. Marler, K. Muir, N. Venketasubramanian, R. Sacco, A. Shuaib, P. Teal, N. G. Wahlgren, S. Warach, and C. Weimar.
